# Supplementary material for: End Sequence Analysis Toolkit (ESAT) expands the extractable information from single-cell RNA-seq data
Source: Genome Res. 2016 Oct;26(10):1397–410. doi: 10.1101/gr.207902.116 (PMC5052061; doi:10.1101/gr.207902.116)
Supplement: Supplemental Material [file supp_gr.207902.116_Supp_Materials.tar.gz › ESAT-esat_v0.1/doc/html/namespacebroad_1_1core_1_1siphy.html]

ESAT: Package broad.core.siphy


ESAT


- Main Page
- Related Pages
- Packages
- Classes
- Files

- Packages

All Classes Namespaces Files Functions Variables Enumerator Pages

- broad
- core
- siphy

Packages |
Classes

Package broad.core.siphy

|  |  |
| --- | --- |
| Packages | |
| package | tools |
|  | |

|  |  |
| --- | --- |
| Classes | |
| class | ConservationUtils |
|  | |
| class | ConservedPWMScanner |
|  | |
| class | EvolutionaryModel |
|  | |
| class | EvolutionaryModelParameters |
|  | |
| interface | Fit |
|  | |
| class | PhastconsReader |
|  | |
| class | PiHMM |
|  | |
| class | PositionWeightMatrixModel |
|  | |
| interface | ScaledWindowParserListener |
|  | |
| class | StationaryDistributionIO |
|  | |
| class | TreeScaler |
|  | |
| class | TreeScalerIO |
|  | |
| class | UnableToFitException |
|  | |


---

Generated on Fri Aug 15 2014 15:10:56 for ESAT by  

 1.8.7
